# Pulpotomy versus root canal treatment in permanent teeth with spontaneous pain: comparable clinical and patient outcomes, but insufficient evidence

**DOI:** 10.1038/s41432-023-00878-4

**Published:** 2023-05-15

**Authors:** Kelvin I. Afrashtehfar, Carlos A. Jurado, Dunia Al-Hadi, Krishna P. Shetty

**Affiliations:** 1 Assistant Professor and Director of the Evidence-Based Practice Unit, Ajman University College of Dentistry, Ajman City, UAE; 2 Visiting Research Professor, Department of Reconstructive Dentistry and Gerodontology, School of Dental Medicine, University of Bern, Bern, Switzerland; 3 Associate Professor, Department of Prosthodontics, The University of Iowa College of Dentistry and Dental Clinics, Iowa City, IA USA; 4 Lecturer in Endodontology, Ajman University College of Dentistry, Ajman City, UAE

**Keywords:** Dental treatments, Endodontics

## Abstract

**Design:**

A systematic appraisal and statistical aggregation of primary studies.

**Data sources:**

Scopus/ELSEVIER, PubMed/MEDLINE, Clarivate Analytics’ Web of Science (i.e., Web of Science Core Collection—WoS, Korean Journal Database—KJD, Russian Science Citation Index—RSCI, SciELO Citation Index—SCIELO), and Cochrane Central Register of Controlled Trials (CENTRAL) via the Cochrane Library.The complementary searches consisted of OpenGrey, Google Scholar (first 100 returns), Networked Digital Library of Theses and Dissertations, Open Access Theses and Dissertations, DART-Europe E-theses Portal—DEEP, Opening access to UK theses—EThOS.

**Study selection:**

Human clinical trials studies in English language with at least 10 patients with mature or immature permanent teeth with pulpitis characterized by spontaneous pain in each arm (i.e., root canal treatment [RCT] and pulpotomy) at the end of the study, comparing the patient- (Primary: survival, pain, tenderness, swelling assessed by clinical history, clinical examination, and pain scales; Secondary: tooth function, need for further intervention, adverse effects; OHRQoL using a validated questionnaire) and clinical-reported outcomes (Primary: emerging apical radiolucency as per intraoral periapical radiograph or limited FOV CBCT scan; Secondary: radiological evidence of continued root formation and presence of sinus tract).

**Data extraction and synthesis:**

Two independent review authors conducted study selection, data extraction and risk of bias (RoB) assessment and a third reviewer was consulted for solving disagreements. When insufficient or absent information, the corresponding author was reached out to for further explanation. The Cochrane RoB tool for randomized trials (RoB 2.0) was evaluated the quality of studies.The meta-analysis was performed on a fixed-effect model to estimate pooled effect size such as odds ratio (OR) and 95% confidence intervals (CIs) were performed using the R software. The quality of evidence assessed by the Grading of Recommendations, Assessment, Development and Evaluations (GRADE) approach (GRADEpro GDT: GRADEpro Guideline Development Tool [software], McMaster University, 2015).

**Results:**

Five primary studies were included. Four studies referred to a multicentre trial assessing postoperative pain and long-term success rate after pulpotomy compared with one-visit RCT in 407 mature molars. The other study was a multicentre trial assessing postoperative pain in 550 mature molars treated with pulpotomy and pulp capping with the calcium-enriched mixture (CEM), pulpotomy and pulp capping with mineral trioxide aggregate (MTA) and one-visit RCT. Both trials primarily reported first molars from young adults. When looking at the results of postoperative pain, all the trials included had a low RoB. However, when evaluating the clinical and radiographic outcomes of the included reports, it was determined that there was a high RoB. The meta-analysis found that the likelihood of experiencing pain (i.e., mild, moderate, or severe) at the 7th postoperative day was not affected by the type of intervention (OR = 0.99, 95% CI 0.63–1.55, I^2^ = 0%).The study design, risk of bias, inconsistency, indirectness, imprecision, and publication bias domains were used to grade the quality of evidence for postoperative pain between RCT and full pulpotomy, resulting in a ‘High’ grade. In the first year, clinical success was high for both interventions, with a rate of 98%. However, the success rate declined over time, with pulpotomy showing a 78.1% success rate and RCT showing a 75.3% success rate at the 5-year follow up.

**Conclusions:**

This systematic review was limited by the inclusion of only two trials, indicating a lack of sufficient evidence to draw definitive conclusions. Nonetheless, the available clinical data suggests that patient-reported pain outcomes do not differ significantly between RCT and pulpotomy at Day 7 postoperatively, and that the long-term clinical success rate of both treatments is comparable, as demonstrated by a single randomized control trial. However, to establish a more robust evidence base, additional high-quality randomized clinical trials, conducted by diverse research groups, are needed in this field. In conclusion, this review underscores the insufficiency of current evidence to draw solid recommendations.

## A Commentary on

**Tomson P L, Vilela Bastos J, Jacimovic J, Jakovljevic A, Pulikkotil S J, Nagendrababu V**.

Effectiveness of pulpotomy compared with root canal treatment in managing non-traumatic pulpitis associated with spontaneous pain: A systematic review and meta-analysis. *Int Endod J* 2022; 10.1111/iej.13844.

**GRADE Rating:** 

## Commentary

Untreated caries in permanent teeth is a prevalent global health condition that can lead to inflammation of the pulp, resulting in reversible or irreversible pulpitis, which may or may not cause pain^1,2^. In cases of irreversible pulpitis (IP), the clinician is typically limited to either root canal treatment (RCT) or extraction as treatment options. While extraction is always effective, RCT can also be highly successful if performed correctly. However, RCT is a technically challenging and time-consuming procedure that weakens the tooth’s structure and leaves it more vulnerable to infection and caries^3–7^. These concerns highlight the need for less invasive and biologically based treatment options.

Vital pulp treatment is now considered a reliable treatment even in cases with carious pulp exposure^1,8^. A pulpotomy is a technique used to preserve pulp tissue and has been revisited as a permanent treatment modality, especially in cases of irreversible pulpitis. The use of calcium silicate cements has further increased the success rates of pulpotomy in such cases. High short-term success rates (i.e., 92% at 2-yrs) have been reported for both partial^9,10^ and full pulpotomy^11^. The removal of some or all the coronal pulp tissue is a clinical approach to manage irreversible pulpitis by eliminating inflamed tissue, relieving pain, and inducing hard tissue barrier using a calcium silicate cement that stimulates the pulp’s natural reparative mechanisms^12^.

Therefore, the appraised systematic review by Tomson et al. (2022) aimed to evaluate whether a pulpotomy (partial or full) could result in better patient and clinical reported outcomes compared to root canal treatment (RCT) in permanent teeth with pulpitis characterized by spontaneous pain. The review included five studies, three of which reported longer-term data on the same cohort of patients at different time points and two clinical trials with shorter-term outcomes. The results suggested that patients experience similar levels of pain postoperatively, irrespective of whether they are treated with RCT or full pulpotomy. The success rates for both interventions were high at 12-month follow-up and were reduced at 24 and 60-month follow-up, but there was no significant difference in success between both interventions. These findings are supported by previous reviews that reported that pulpotomy with calcium silicate cements is an effective treatment option for patients with pulpitis characterized by spontaneous pain managed by vital pulp therapy (VPT)^13–17^.

The review identified the benefits of pulpotomy, including its reduced aggressiveness, ability to maintain pulp functions, and improved cost-effectiveness compared to RCT. However, the review notes that the number of studies on this topic is limited, which makes it challenging to establish a strong evidence-based recommendation. Thus, no publications bias was performed. The study finds that patients experience similar levels of postoperative pain with both pulpotomy and RCT. Additionally, patients with apical periodontitis or periodontal ligament (PDL) widening had significantly more postoperative pain regardless of the treatment modality. The review identifies the limitations of the studies, including the fact that RCTs performed in a single visit are not typical in everyday dental practice. Furthermore, 33% of patients were lost to follow-up in one study^18^, which may impact the strength of the results.

The strength of the results is weakened by the fact that the longer-term outcomes are derived from the analysis of only one cohort of patients at different postoperative time points^18–21^. Additionally, the use of calcium silicate cement, namely calcium-enriched mixture (CEM) cement, was limited to the country of manufacture. Furthermore, the study does not indicate any quality assurance for the general standard of treatment performed in either arm. However, the review acknowledges that the study was performed in a primary care setting, making it possible to extrapolate the results to a setting where most of the treatment for pulpitis with spontaneous pain is performed.

Future research is required to establish a more robust evidence-based clinical practice for managing pulpitis characterized by spontaneous pain. It is necessary to conduct more clinical trials to assess the effectiveness of different agents to control hemostasis, cleanse the cavity/exposed pulp, or interface with the pulp. Further research is required to determine the optimal follow-up period for treatment outcomes, which should consider the high loss of patients during the long-term follow-up. Establishing standardized protocols for RCT and pulpotomy that consider patients with different periapical conditions is also essential. Additionally, future research should assess the cost-effectiveness of pulpotomy and RCT in different healthcare settings. Overall, more research is necessary to inform solid evidence-based clinical recommendations for managing pulpitis with spontaneous pain.

To sum up, this well-conducted systematic review with quantitative analysis concluded that pulpotomy is a viable alternative to RCT. However, more high-quality clinical studies are required to provide reliable clinical practice recommendations.

Practice points

- Insufficient evidence exists to establish clear differences between root canal treatment and pulpotomy in terms of patient-reported pain at day 7 postoperatively and long-term clinical success rate.
- If the comparative effectiveness of pulpotomy as a definitive treatment modality was demonstrated to be on par with that of root canal treatment on permanent teeth with spontaneous pain, it would retain a vital pulp, obviate the need for root canal treatment, and lower treatment cost and duration.
